# Direct and averted gaze modulate the event-related brain responses to social exclusion signals

**DOI:** 10.1038/s41598-025-97840-4

**Published:** 2025-04-18

**Authors:** Yu-Fang Yang, Xu Fang, Michael Niedeggen

**Affiliations:** Division of Experimental Psychology and Neuropsychology, Department of Education and Psychology, Freie Universität Berlin, Berlin, Germany

**Keywords:** Social exclusion, Eye direction, P300, Event-related potentials, Cyberball paradigm, Human behaviour, Neurophysiology

## Abstract

Social exclusion is a negative experience in social interaction which can be modulated by the perceived eye gaze. In our Cyberball study, we asked how gaze direction – direct versus averted – affects the processing of social exclusion by using electroencephalogram (EEG) methods. Participants encountered neutral gazes in the inclusion phase (Block1) and then either direct or averted gazes during the exclusion phase (Block2). We measured self-reports on Need-Threat Questionnaires (belonging, control, meaningful existence, self-esteem) and negative mood, along with event-related potentials (ERPs), specifically the P3 component (P300) - an indicator of expectation violation. Findings demonstrated that the P3 amplitude showed differential changes between gaze conditions during the transition from inclusion to exclusion, with direct gaze eliciting stronger neural responses. This difference signals that direct gaze enhances the expected participation, thus sensitizing participants for exclusionary signals. Simultaneously, self-reports indicated that the transition evoked weaker negative emotions in the direct gaze group and stronger negative emotions in the averted gaze group, signalling that direct gaze also serves as an affiliative signal. These results illuminate how gaze directions shape cognitive and emotional reactions to social exclusion. However, further research is necessary to unravel these effects amidst other non-verbal cues and fully comprehend their impact on neural and behavioural responses to social exclusion.

**Supplementary Information:**

The online version contains supplementary material available at 10.1038/s41598-025-97840-4.

## Introduction

Imagine engaging in a conversation where the speaker avoids making eye contact with you. Such a subtle cue can evoke feelings of exclusion, and prompt questioning about one’s social standing. Social exclusion, characterized by being disregarded or rejected in social interactions, adversely affects psychological well-being and satisfaction of fundamental social needs. It is typically assessed using the Need-Threat Questionnaires (NTQ) that quantify elements such as belonging, control, self-esteem, and meaningful existence^1,2^. It has been equated with experiencing physical pain in terms of its immediate detectability, and can lead to alterations in behavioural choices and impairments in emotional and cognitive domains^3–5^.

Eye gaze, a subtle non-verbal signal, plays a crucial role in human social interaction. It is a potent modulator of social cognition, conveying intent^6,7^, emotion^8,9^ (see review^10^), and attention^11^. In situation of social inclusion or exclusion, the direction of gaze can considerably influence one’s perception of the interaction^12,13^. For instance, Wirth and colleagues (2010) reported that averted eye gaze led to feelings of ostracism, reduced self-esteem, and increased temptations to act aggressively^14^.

The Cyberball paradigm, a virtual ball-tossing game, is commonly used to study social ostracism^15–19^. During the game, the participant is represented by an avatar who is connected to two other avatars, who are purportedly human co-players. The co-players either pass the ball to the participant or toss the ball exclusively among themselves, creating the feeling of being excluded. Such exclusionary actions contradict the participant’s subjective expectation of equal participation and provoke psychological distress^21,22^. Brief exposures to exclusion, as short as three minutes, have been found to significantly impact mood and diminish satisfaction across four fundamental psychological needs: belonging, control, self-esteem, and meaningful existence^23^, thus underscoring the paradigm’s efficacy in eliciting substantial emotional and cognitive responses to social exclusion^15,17^.

Studies have demonstrated that social exclusion can heighten attention to social cues, such as gaze direction. Excluded individuals are more attentive to the gaze direction of others, which affects their perception and interpretation of social interactions^13^. Research on gaze and social exclusion has revealed two distinct processes. Previous work has primarily focused on how experiencing exclusion influences subsequent gaze processing, showing that excluded individuals become more sensitive to direct gaze as a potential signal for social reconnection^12,14,24–28^. The fundamental connection between gaze and belonging lies in gaze’s role as a primary social signal - averted gaze communicates social withdrawal and triggers the same neural threat-detection mechanisms that respond to explicit exclusion, while direct gaze satisfies our basic need for social connection by signalling potential for interaction^8,10^.

Most prior research has focused on gaze processing after exclusion events, rather than examining how gaze direction itself shapes the immediate experience of social exclusion^24,25,28^. Moreover, social exclusion affects the perception of the cone of gaze, with excluded individuals perceiving a narrower range of gaze directions as direct^26^. Ostracized individuals interpret sustained averted gaze as an additional sign of social exclusion, leading to feelings of disengagement and a reduced motivation to interact with others. This is supported by findings that individuals who experience ostracism exhibit altered processing of gaze signals, expressing itself in a reduced accuracy in identifying the gaze direction^12^. These findings support the notion that lack of eye contact provides a strong non-verbal social cue to indicate the feeling of rejection and neglect as the responses of ‘not talking’ and ‘avoiding all contact’^29^. On the contrary, sustained and direct eye contact indicates attention, affection, love, and other positive affective intentions^14,30^. Sustained eye contact can enhance the perception of others’ intentions and interests^10,11^, and elicit positive feelings across different cultures^31^. In Capellini et al.’s (2017) study^24^, direct gaze was defined as indicating social engagement and inclusion, where the gaze was directed towards the participant’s avatar. Conversely, ‘averted gaze’ is defined as indicating social disengagement and exclusion, serving as a non-verbal cue for social exclusion.

Furthermore, gaze information’s role in social re-affiliation has been noted, with ostracized individuals seeking direct gazes as cues for inclusion^27^. Additionally, social inclusion, but not exclusion, delays attentional disengagement from direct gazes, indicating a complex interaction between gaze direction and social perception^25^. While existing literature underscores gaze direction’s influence on social inclusion and exclusion perceptions^12–14,25,26^, its manipulation within such contexts remain underexplored. Studies have diverged in their approaches; for example, Capellini et al. (2017) conducted a gaze-cueing task following the exclusion event in Cyberball, examining how individuals orient their attention based on the gaze direction of others^13^. Similarly, Bossi et al. (2018) manipulated gaze direction after the exclusion event to assess its impact on the processing of social information and found that social exclusion increases sensitivity to acceptance signals, leading to reduced accuracy in identifying the direction of gaze^12^. These studies illustrate two distinct approaches in gaze-exclusion research. First, experiencing social exclusion can alter how individuals process and respond to gaze cues, as demonstrated by Bossi et al. (2018), where exclusion increased sensitivity to acceptance signals and affected gaze direction identification^12^. Second, and central to our current investigation, is how gaze direction itself serves as a social signal during ongoing interactions. This distinction helps clarify that while earlier work examined gaze processing as an outcome of exclusion, we investigate gaze as a concurrent social signal that may shape how exclusion is experienced and processed.

While behavioural data have yielded valuable insights, the integration of psychophysiological measures, such as electroencephalogram (EEG), offers a more immediate and objective evaluation of responses to social exclusion in the Cyberball paradim^22,32,33^. The P3 component of event-related potentials (ERPs), a prominent positive deflection in the EEG signal occurring typically between 300 and 600 ms after stimulus onset, is particularly insightful for its correlation with attention and cognitive processing^34,35^. More importantly, the P3 component is a reliable indicator for the processing of exclusionary signals in the Cyberball paradigm – as an indicator of expectation violation in the context of social inclusion, see Vanhollebeke et al.^18^ for a recent review. More specifically, the P3 amplitude is enhanced by a transition from inclusion to exclusion, and this effect is primarily due to a violation of subjective expectation on involvement^36–38^. This account has been further supported by recent studies showing enhanced P3 responses to concurrent social threats, suggesting a common cognitive system for processing violations of social expectations^36,39^.

Psychophysiological research, which often employs extended stimulus sequences, enables the observation of P3 amplitude fluctuations throughout exclusionary scenarios. Changes in P3 amplitude have previously been associated with continuous readjustment in response to unexpected events. Corresponding changes in a Cyberball game can be viewed as a compensatory effort in response to a perceived aversive situation^40^. Studies have shown that both transitions, from inclusion-to-exclusion and from control-to-a-loss-of-control, result in an increase in P3 amplitude^21,41^. These transitions indicate a disruption in the participant’s expectation, eliciting larger P3 responses as they attempt to adjust their expectations to the new situation. As participants continue to experience the exclusionary setting, their P3 amplitudes gradually decrease, reflecting a continuous readjustment process in response to unexpected events.

Considering the importance of gaze direction as a non-verbal social cue, the observed changes in P3 amplitude serve as a marker of expectancy violations in social contexts and may reflect the level of involvement and affiliative intent. The self-report data from the NTQ adds critical information to the online measurement of P3. Although not necessarily correlated^36,42^, the retrospective reports signal the affective changes induced by the transition to exclusion^22,33,37,43^, reflecting stimulus evaluation. This will add information on the psychological effects of the exclusionary experience not necessarily provided in the ERPs. This is particularly relevant in our study, as we explore how gaze direction may influence these expectations and subsequent experiences of social exclusion. In addition to P3, analysis will also focus on the questionnaire data (NTQ) and negative mood to examine whether gaze information will also affect the self-reported feeling of exclusion and mood.

In summary, our study aims to extend the understanding of non-verbal social cues by examining the effects of direct and averted gaze on the cognitive and emotional processing of social exclusion within the Cyberball paradigm, using EEG to measure brain activity. The measures used in the study included the NTQ to assess perceived exclusion and a negative mood questionnaire to evaluate emotional responses. This integrated approach allows us to interpret the neural data within the context of participants’ self-reported feelings of exclusion and negative affect.

## Current study

To investigate how gaze direction influences social exclusion processing, we manipulated co-player avatars’ gaze direction within the Cyberball paradigm. Our study aimed to address two key research questions: (1) how gaze direction affects expected social participation, and (2) how does P3 amplitude reflect the violation of expected ball reception during social exclusion? To examine these hypotheses, we examine how unexpected changes in ball reception elicit P3 responses, with larger P3 amplitudes indicating stronger violations of participants’ expectations to receive the ball from co-players. To be more precise, we hypothesized that direct gaze would lead to lower perceived exclusion and negative mood but increased P3 amplitudes, reflecting stronger expectancy violation (*Hypothesis 1*). Conversely, averted gaze was predicted to result in higher reported exclusion (i.e., stronger perceived threat to fundamental needs and increased negative mood) but reduced P3 amplitudes, indicating anticipatory adjustment to exclusion (*Hypothesis 2*). Participants were divided into two groups: one encountering avatars exhibiting direct gaze, and another encountering avatars displaying averted gaze. In Block 1, participants experienced neutral gaze and received an equitable 33% share of ball tosses, establishing a baseline of inclusion. In Block 2, social exclusion was simulated as co-players reduced ball-tossing to 23%, with gaze direction adjusted to either direct or averted.

## Results

### Behavioural data

#### Manipulation check: Ball reception

The first analysis assessed the perceived frequency of ball receptions across the two blocks, as reported by participants’ post-experiment. Participants reported perceiving a reduced frequency of ball reception in Block 2 (exclusion) compared to Block 1 (inclusion), see Table 2. An ANOVA substantiated a significant main effect of Block, *F*(1, 54) = 92.96, *p* < .001, *η*_*p*_^2^ = 0.633, Cohen’s *d* = 1.64, indicating that participants noted less frequent ball receptions during Block 2, 95% CI [17.88, 22.48] than Block 1, 95% CI [33.86, 39.81]. Neither the main effect of the Group, *F*(1, 54) = 0.03, *p* = .873, *η*_*p*_^2^ < 0.001, nor the interaction with Block, *F*(1, 54) = 0.60, *p* = .442, *η*_*p*_^2^ = 0.011, reached significance, suggesting a consistent recognition of reduced ball reception across groups.

#### NTQ

Our analysis sought to elucidate the influence of eye gaze direction on social exclusion processing as indicated by the NTQ scales (see Table 1). The multivariate main effect for Group was not significant, *F*(1, 54) = 0.825, *p* = .368, *η*_*p*_^2^ = 0.015, indicating no discernible difference between the Direct and Averted gaze groups across the combined dependent measures. However, the multivariate main effect for the within-subjects factor was significant, *F*(3, 162) = 5.732, *p* < .001, *η*_*p*_^2^ = 0.096, denoting significant differences across the four dependent measures. Subsequent univariate ANOVAs revealed no significant effect of Group on any individual dependent measure: belonging, *F*(1, 54) = 0.685, *p* = .412, *η*_*p*_^2^ = 0.01; meaningful existence, *F*(1, 54) = 1.972, *p* = .166, *η*_*p*_^2^ = 0.04; self-esteem, *F*(1, 54) = 0.15, *p* = .7, *η*_*p*_^2^ < 0.001; or control, *F*(1, 54) = 0.082, *p* = .776, *η*_*p*_^2^ < 0.001.

**Table 1:** Descriptive statistics of self-report data post-cyberball paradigm.

	Direct gaze (*n* = 28)	Averted gaze (*n* = 28)
	M ± SD	M ± SD
NTQ: belonging	− 0.73 ± 0.78	− 0.94 ± 0.78
NTQ: control	− 0.38 ± 0.77	− 0.44 ± 0.78
NTQ: meaningful existence	− 0.36 ± 0.96	− 0.73 ± 0.96
NTQ: self-esteem	− 0.35 ± 0.92	− 0.45 ± 0.92
Negative mood	0.66 ± 2.80	2.34 ± 3.22

The table presents mean scores and standard deviations (M ± SD) for the need threat questionnaire (NTQ) scales and negative mood, following the cyberball paradigm. Negative NTQ values suggest a stronger perception of threat or exclusion. Scores are divided by the gaze direction groups: direct gaze and averted gaze, with 28 participants in each group.

#### Negative mood

Conversely, the negative mood score was elevated (more negative) in the averted gaze group relative to the direct gaze group. A significant difference emerged in negative mood between groups, *t*(54) = 2.08, *p* = .042, Cohen’s *d* = 0.57, 95% CI [0.02, 1.11], with participants in the averted gazes group reporting more negative feelings. It is crucial to underscore that the assessment of negative mood was conducted independently of the NTQ, utilizing the Positive and Negative Affect Schedule (PANAS) questionnaire to specifically evaluate negative mood.

### ERP data

#### Transition from inclusion to exclusion (effect of blocks)

**Fig. 1:**
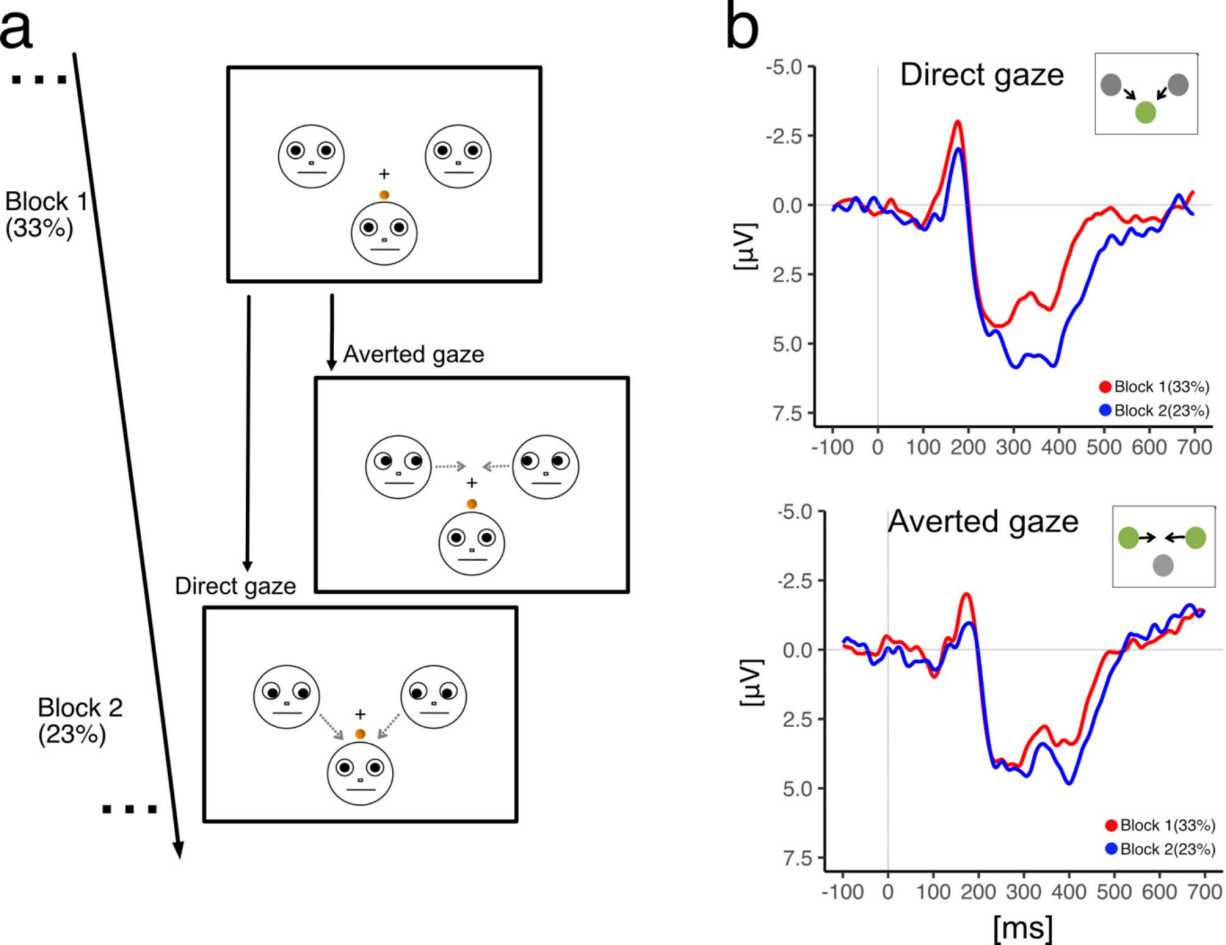
Schematic representation and ERP response of the cyberball paradigm. (**a**) cyberball game depiction. The main display presents three avatars: two representing virtual co-players situated at the top left and top right, and one representing the participant at the centre bottom. In Block 1, the co-players’ avatars exhibited a neutral gaze directed forward. However, during Block 2, a modification in the co-players’ gaze was introduced. For the direct gaze group, participants encountered direct gazes from the virtual co-players, while the averted gaze group participants observed avatars with sideward gazes. In Block 1, a 33% ball possession was evenly distributed among players. This possession experienced a minor reduction to 23% in Block 2. The gray dashed lines in Block 2, indicating gaze direction, are merely illustrative and were absent during the actual experiment. (**b**) ERP response during ball possession events. presented here are grand-average ERP patterns over a fronto-central region of interest (Fz, Cz, F3, F4). These are time-locked to the event of receiving the ball. Displayed waveforms differentiate between the conditions (Block 1 in red and Block 2 in blue) and are further categorized by the gaze orientation (direct vs. averted).

Analyses of the event ‘self’ (when the participant receives the ball) were conducted. The grand average ERPs exhibited a sustained positive peak between 320 and 400 ms, see Fig. 1b. This peak was more pronounced during the second block (i.e., exclusion) compared to the first block (i.e., inclusion), indicating a greater neural response to the receipt of the ball during this period, irrespective of the gaze direction of the avatars, see Table 2.

**Table 2:** Descriptive statistics of estimated frequency (%) ball reception and ERP data across blocks.

Measure/block		Block 1 Mean(SD) 95%CI		Block 2 Mean(SD) 95%CI	
Estimated frequency (%) ball reception	Direct gaze	36.00 (10.79)	[33.22, 42.14]	20.68 (9.51)	[24.20, 17.16]
	Averted gaze	37.68 (12.04)	[33.21, 42.13]	19.68 (8.15)	[12.66, 22.70]
P3 (µV)	Direct gaze	3.49 (3.17)	[2.32, 4.67]	5.51 (3.35)	[4.27, 6.75]**
	Averted gaze	3.11 (2.62)	[2.14, 4.08]	2.85 (2.85)	[2.72, 4.83]

This table presents the mean values along with the standard deviation (in parentheses) and the 95% confidence intervals (in square brackets) for the P3 across the two blocks. ERP measurements are based on the frontal-central cluster of electrodes (Fz, Cz, F3, F4). **Indicates *p* < .01 between Block 1 and Block 2 for the Direct gaze group.

**Fig. 2:**
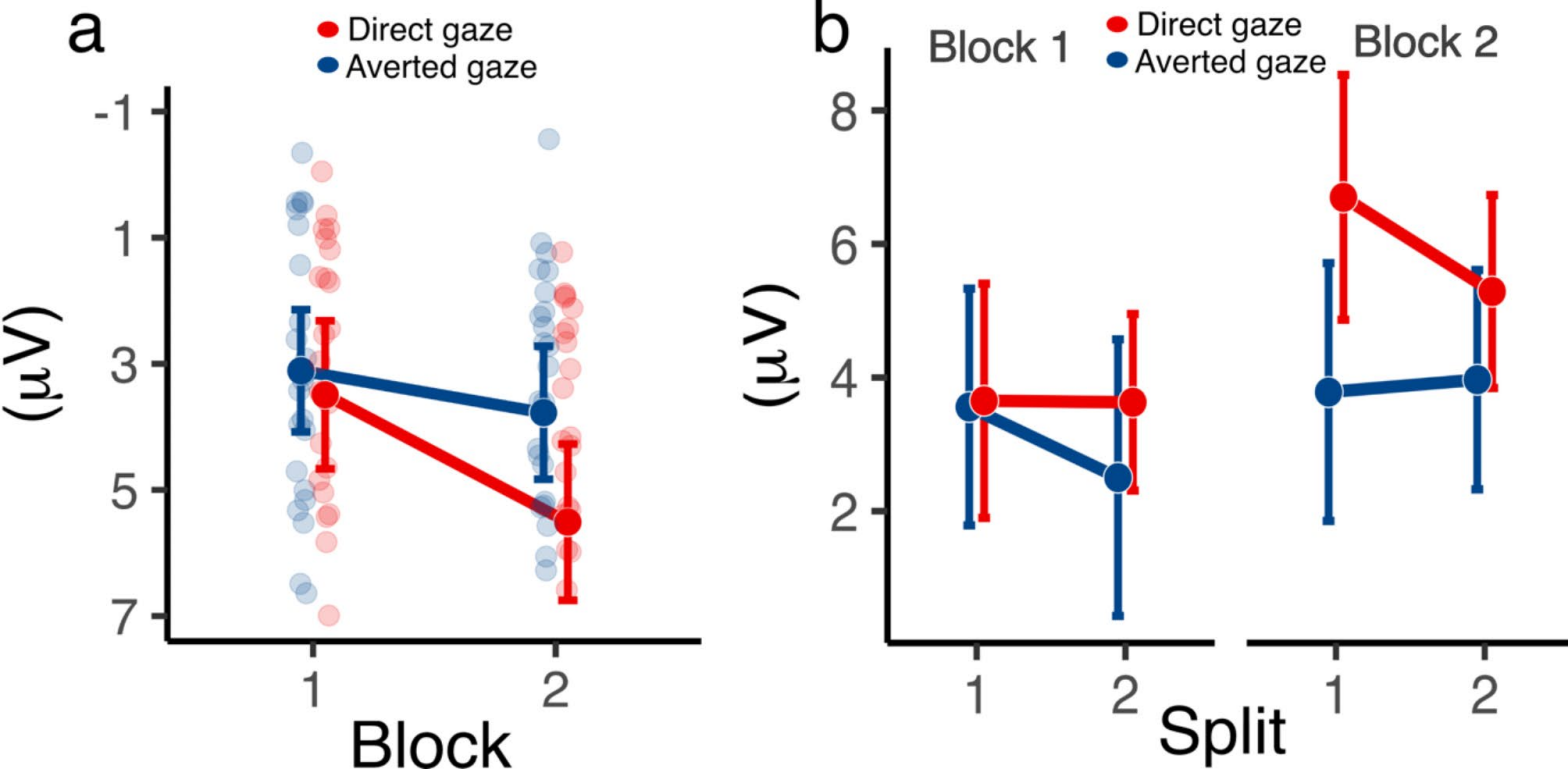
Depiction of average P3 amplitudes on the Fz, Cz, F3, and F4 channels for the 320–400 ms time window. (**a**) Line plots for Block 1 and Block 2 differentiating between direct gaze (in red) and averted gaze (in blue). Each light dot represents individual subject data. The error bars indicate 95% confidence intervals. (**b**) Line plots separated by block and split (first vs. second half of the experimental block), grouped by gaze type (direct vs. averted). Error bars represent 95% confidence intervals.

The increase in amplitude in Block 2 resulted in a significant main effect of Block, *F*(1, 54) = 20.61, *p* < .001, *η*_*p*_^2^ = 0.276. This indicates that there was a statistically significant difference in the amplitude of P3 between the two blocks, with the amplitude greater in Block 2 (*M* = 4.64 µV, 95% CI [3.81, 5.48]) compared to Block 1(*M* = 3.30 µV, 95% CI [2.54, 4.06]. The main effect of Group was not significant (*F*(1,54) = 2.00, *p* = .163, *η*_*p*_^2^ = 0.036). However, a significant Group × Block interaction emerged, *F*(1,54) = 5.23, *p* = .026, *η*_*p*_^2^ = 0.088, indicating differential P3 responses between gaze conditions during the transition from inclusion to exclusion, Fig. 2a. Post-hoc tests revealed that while the direct gaze group showed a significant increase in P3 amplitude from Block 1 to Block 2 (t(54) = −3.234, *p* = .0109). However, no significant change was observed for the Averted Gaze group (t(54) = −1.564, *p* = .484). To further examine the observed interaction, we computed block differences (Δ(Block 2 - Block 1)) to quantify how P3 amplitude changed during the transition from inclusion to exclusion. This analysis confirmed a significant group effect (*F*(1,54) = 5.23, *p* = .026, *η*_*p*_^2^ = 0.09), indicating that gaze direction influenced the degree of P3 modulation over time. Post-hoc comparisons revealed that the direct gaze group (M = 5.51, SD = 3.35) exhibited a greater increase in P3 amplitude compared to the averted gaze group (M = 3.78, SD = 2.85), t(54) = 2.089, *p* = .040.

### Split-half analysis: adaptation effects within blocks

The split-half analysis, intended to explore adaptation to social exclusion, showed significant changes in P3 amplitude within the direct gaze group from the first to the second block, *p* = .0001. Consistent with prior studies^37,41,44^, decrease in P3 amplitude was expected across blocks (from 33 to 23%). This analysis, crucial for uncovering cognitive and emotional responses to social exclusion, was conducted on a data subset from participants with over 20 trials per block (as delineated in the Method section).

The split-half analysis was included to explore potential adaptation effects within blocks, providing a more detailed examination of how P3 amplitudes change over time within each block. Previous Cyberball studies^21,41^ have shown that the sustained processing of an exclusionary signal might be processed differently over time. This process can be uncovered by a split-half analysis. The ANOVA revealed no significant three-way interaction between gaze direction, block, and split (F(1, 38) = 1.96, *p* = .170, ηp^2^ = 0.049), indicating that the adaptation process was not differentially expressed between gaze conditions. While the direct gaze group exhibited a trend of increased P3 amplitude during exclusion, it did not significantly diverge from the averted gaze group.

ANOVA results confirmed a significant Block effect: P3 amplitudes were notably higher in the second block (95% CI [3.84, 6.34]) than in the first block (95% CI [2.18, 4.58]), *F*(1, 38) = 19.56, *p* < .001, *η*_*p*_^2^ = 0.340, as detailed in Table 3. Additionally, a significant interaction between group and block, *F*(1, 38) = 4.34, *p* = .044, *η*_*p*_^2^ = 0.102, was observed. Post hoc analysis showed a larger P3 amplitude in the second block for the direct gaze group (95% CI [4.34, 7.65]) compared to the first block (95% CI [2.11, 5.64]), *p* = .0001, suggesting more pronounced exclusion effects compared to the averted gaze group (Fig. 2b). No main effect for split (*F*(1, 38) = 1.70, *p* = .201, *η*_*p*_^2^ = 0.043), or for group (*F*(1, 38) = 1.82, *p* = .185, *η*_*p*_^2^ = 0.046), *η*_*p*_^2^ was observed. These findings prompt a re-evaluation of our initial interpretation and highlight the need for further investigation into the nuanced effects of gaze direction on social exclusion processing.

**Table 3:** Mean P3 amplitudes (µV) ± standard deviations across blocks and block halves (split). This table shows the mean P3 amplitudes (in µV) as means ± standard deviations for direct and averted gaze groups during the first and second halves (splits) of blocks 1 and 2.

Group	Block 1		Block 2	
	1st half	2nd half	1st half	2nd half
Direct gaze	3.65 ± 4.89	3.63 ± 3.67	6.70 ± 4.60	5.30 ± 4.00
Averted gaze	3.56 ± 4.10	2.50 ± 4.51	3.78 ± 4.72	3.97 ± 4.72
Direct gaze	3.64 ± 3.76		5.99 ± 4.01	
Averted gaze	3.03 ± 4.03		3.88 ± 3.71	

## Discussion

This study explored how gaze direction influences the perception of social exclusion during a virtual Cyberball game, using EEG and self-report measures to assess how gaze affects cognitive processing during exclusion. Our results indicate that direct gaze, compared to averted gaze, enhances P3 responses. This effect appears independent of the duration of the exclusion phase. Regarding self-reports, the transition from inclusion to exclusion did not significantly alter the perceived sense of belonging between the two groups. However, participants experienced greater negative mood when exclusion was paired with averted gaze rather than direct gaze provided by the avatars of the co-players. These results will be discussed in the following sections.

At first, the modulation of the P3 effect by gaze condition will be discussed. Our results confirm that P3 amplitude elevation accompanies the shift from inclusion to exclusion, suggesting even nuanced changes in ball reception can invoke a P3 effect^32,38^. This effect has been previously linked to expectancy violations, specifically when social inclusion expectations are not met, triggering enhanced cognitive processing of exclusionary cues^14,45^. The P3 increase is influenced not only by probability of ball reception but also by social cues such as gaze direction. Direct gaze from co-players’ avatars significantly accentuated the P3 effect during exclusion compared to averted gaze, highlighting the role of social attention in modulating expectancy violations. These observations are consistent with expectancy-violation accounts models - direct gaze can be assumed to induce a higher level of initial expectations of inclusion, and consequently social exclusion onset was less expected^21^.

The different expectancies observed between the gaze conditions correspond to prior findings suggesting that individuals adjust their social expectations depending on the perceived likelihood of inclusion or exclusion^2,14,38^. When direct gaze signals potential engagement, it amplifies neural responses^8,46^. In contrast, averted gaze serves as an exclusionary cue, mitigating expectancy violations and prompting lower P3 responses^47^. This mechanism might reflect an adaptive process in which social cues dynamically shape anticipatory cognitive processing. Such strategies are characteristic of the ‘inconsistency compensation’ mechanism, wherein individuals attempt to reconcile discrepancies between anticipated and actual social outcomes^42,48^. Prior research indicates that excluded individuals might engage in nonverbal behaviours, such as eye contact, to restore social bonds and mitigate the psychological distress of exclusion^49^.

The modulation of the P3 effect – described above – is based on the response averaged across the set. A further analysis tested whether the difference of the P3 effect between groups might depend on temporal dynamics, i.e. whether these differences might change over time. Prior research^21,37^ indicated that a reduction in P3 amplitude during the exclusion phase can be related to an adaptation to the anticipated lack of participation. However, the split-half analysis did not show a corresponding adaptation process triggered by differential gaze information (Fig. 2b).

Based on the finding that differences in the P3 amplitude between the two groups were already present at the beginning of the exclusionary experience, we suggest that gaze direction modulates cognitive adjustment differently. Direct gaze may sustain initial expectations of inclusion, while averted gaze signals impending exclusion early on, prompting a quicker disengagement from social interaction. However, interpretations of these patterns should be approached cautiously, given the limited dataset and potential issues with statistical power. Future studies should aim to disentangle the distinct contributions of social and spatial cues, specifically gaze direction, to clarify their roles in modulating neural responses to social exclusion. This would provide a more comprehensive understanding of how gaze direction dynamically shapes cognitive adaptation during exclusion.

To assess whether the P3 pattern extended to subjective experience, we examined self-reports of fundamental needs and negative emotion. The P3 effect was assumed to be matched by the self-reports, but the expected enhancement of both fundamental needs and negative emotion in the direct gaze condition was not observed. The effects of the level of self-reports contradicted our hypothesis: the expected enhancement of both, fundamental needs and negative emotion, in the direct gaze condition was not observed. Instead, exclusion heightened negative mood more prominently in the averted gaze group. We suggest that this pattern of results reflects a greater sensitivity to exclusionary cues when gaze is averted. The experience of averted gaze has been linked to increased feelings of isolation, as demonstrated by studies emphasizing its role in amplifying exclusion-related affective responses^14^. This heightened sensitivity can intensify the perceived sense of disconnection, limiting opportunities for reconnection and reinforcing the perception of others as distant.

Although negative mood increased in the averted gaze condition compared to the direct gaze condition, this did not translate into significantly different NTQ scores between groups. One might assume a higher validity of the PANAS-based negative mood scale (based in eight items) as contrasted to a single NTQ scale (based on four items), and that the negative mood scale is therefore more sensitive to reflect effects induced by rather small changes in ball reception. The proposed differential sensitivity between PANAS and NTQ scales might be supported by the reflexive stage in Williams’ model^2^: Here, the two processes, threat detection and pain response, have been related to different modules.

According to the Williams model, social exclusion triggers an immediate affective reaction, which serves as an alarm system signalling social threat. The pain-based system that triggers negative affect (measured by PANAS) appears to be more readily activated, possibly serving as an early warning mechanism for potential social threats, while the need-threat system (measured by NTQ) may require more elaborate processing. The exclusion phase involved a reduction to 23% of ball tosses as compared to the 16% typically used in previous studies^37,50^. This mild reduction also leads to a decrease in the expression of the self-reported threat^21^. Interestingly, our findings indicated that the perceived number of ball tosses was not affected by gaze direction manipulation in Block 2 of the Cyberball. This lack of an interaction is likely due to the inherent difficulty in accurately estimating the absolute frequency of ball receptions: High variance has already been observed in previous studies^37^, and interaction effects observed in threat scales are not necessarily reflected in estimated ball possession^50^. Introducing anchoring points might enhance the validity of this scale, providing more consistent results, and should be considered in future research.

The current findings point to a dissociation between the P3 effect and the self-reports measures, suggesting that their relationship cannot be assumed. The observed divergence (P3 effect and NTQ) challenges our initial hypothesis that gaze-induced exclusion would produce parallel effects on both P3 amplitudes and self-reports of social threat. Instead, exclusion under averted gaze conditions enhanced negative mood, but not the P3 effect. In contrast, the P3 responses were primarily elevated in the direct gaze condition, but not negative mood. This discrepancy suggests that neural and self-reported responses to social exclusion may rely on different cognitive processes^36,41,42^.

For example, P3 amplitudes are thought to reflect real-time updates of expectancy violations, capturing immediate shifts in cognitive processing. Research have shown that P3 components can signal exclusion-related expectancy violations even in the absence of the heightened subjective reports, particularly when additional contextual factors are introduced during exclusion^36,41,42^. This effect is further illustrated in paradigms where participants experience a perceived loss of control (e.g., inability to choose which avatar to throw the ball to), with P3 amplitudes remaining elevated while self-reports of belonging threat diminish exclusion^36,41,42^.

In contrast, self-reports, such as those measured by the NTQ and PANAS, represent retrospective evaluations shaped by broader affective and motivational contexts. Studies using the Cyberball paradigm demonstrates that self-reports of exclusion-related distress - such as those assessed through the NTQ - are often influenced by additional factors, including the duration of exclusion and individual differences in emotional coping mechanisms^51^. These findings highlight the These findings highlight the complementary nature of neural and self-report measures in studying social exclusion, as ERPs capture implicit cognitive processes while self-reports reflect retrospective evaluations. However, certain methodological constraints should be acknowledged to contextualize these results.

## Limitations

The limitations of this study warrant caution in result interpretation. The reduced sample size for the second hypothesis, necessitated by stringent EEG pre-processing to ensure accuracy and artifact removal, may have impacted the analysis’s statistical power. Future research should replicate these findings with a broader sample to confirm their robustness. The use of line-drawing faces, while controlling for confounding variables like facial expressions and gender effects, may limit ecological validity. Subsequent studies could enhance validity by incorporating photographic stimuli. Our study’s design aimed to investigate the social implications of gaze direction, yet it simultaneously engaged spatial cues, creating an interpretive complexity^52,53^, While our methodology does not conclusively separate the social from the spatial aspects of gaze, we understand the significance of this distinction for future inquiries. We propose future studies could implement control conditions that isolate facial expression changes, such as variations in the mouth region (e.g., sad vs. happy mouths), without altering interactive patterns in tasks like Cyberball. This could provide a clearer analysis of non-verbal cues’ impact, delineating social from spatial influences. Additionally, our participant pool predominantly consisted of a WEIRD (Western, Educated, Industrial, Rich, Democracies) demographic with an imbalance in gender representation. While measures were taken to mitigate gender disparity, the higher female participation could have influenced outcomes. The potential effects of cultural, educational, and individual differences on our results of gaze directions suggest the need for more diverse samples in future explorations.

## Conclusions

Our research highlights the significant influence of gaze behaviour on social exclusion within the Cyberball paradigm. Averted gaze was associated with increased reports of negative emotions, reinforcing its function as an implicit exclusionary cue, while direct gaze appeared to buffer against the negative affective consequences of exclusion. The P3 responses revealed a heightened sensitivity to expectancy violations in direct gaze conditions, underscoring the cognitive impact of gaze direction during exclusionary experiences.

These findings contribute to the broader understanding of how nonverbal cues – gaze information - influence social exclusion at both neural and subjective levels. While self-reported measures capture retrospective emotional appraisals, ERP data provide insight into real-time cognitive processing of social expectancy violations. The observed dissociation between P3 effects and self-reports emphasizes the need to integrate multiple measures when studying exclusionary processes.

Further research could investigate the interplay between individual personality traits and the observed mechanisms^54^. Additionally, the relevance of these findings in the context of increasing online interactions could be explored, particularly for groups that may experience social communication differently, such as individuals on the autism spectrum. These insights not only advance our understanding of social cognitive processes but also open up potential avenues for fostering resilience in digital communication environments.

## Materials and methods

### Participants

The present study used a mixed-design approach that included both within-participant (“*Block*”) and between-participant (“*Gaze direction*”) factors. Previous ERP studies using the Cyberball paradigm have found that this design is more sensitive in capturing the electrophysiological correlates of the transition from social inclusion to exclusion compared to a between-participant design^5,20,41^. A sample size of 52 participants was calculated a priori using the G*power software^55^ to detect an interaction effect between gaze direction (averted gaze, direct gaze) and within blocks of “inclusion” (33%) to “*partial exclusion*” (23%, referred to as “*exclusion*” from now on) in the Cyberball game. This sample size was necessary to detect a medium effect (*f* = 0.20 according to Cohen’s taxonomy)^56^ with a statistical power of 80% using an *F*-test with the alpha level set at 0.05. Previous Cyberball studies have consistently reported a medium effect size for the modulation of the P3 component^37,38,42^. To ensure a conservative approach in the current study’s novel Gaze Cyberball design, a medium effect size was assumed in the a priori sample size calculation.

A total of 71 participants (44 females, 27 males) were recruited for the study. Of these, 15 participants were excluded due to missing data in the NTQ (*n* = 4), a loss of the raw EEG data (*n* = 1), or due to an excessive number of rejected trials during a rigorous artefact rejection process (*n* = 10, see *Data Analysis* for details). The final sample therefore consisted of 56 participants (36 females, 20 males) aged 19–57 (*M* = 25.2 years, *SD* = 8.0) who reported normal or corrected-to-normal vision and no history of psychiatric or neurological disorders. Participants were randomly assigned to either the direct gaze group (*n* = 28, *M* = 24.6 years ± 5.6, 21 females, 7 males) or the averted gaze group (*n* = 28, *M* = 25.8 years ± 10, 15 females, 13 males). Participants provided written informed consent in accordance with the Declaration of Helsinki and the study was approved by the local ethics committee of the Freie Universität Berlin (No. 037.2022).

### Task and design

The Cyberball paradigm and the following questionnaires were implemented in Python using PsychoPy2, v1.85.6^57^. Prior to the experiment, participants were told that they were being part of a study testing visual imagination capabilities^37,41,42^. To support this cover story, participants first completed a short questionnaire about their visual imagination ability (Vividness of Visual Imagery Questionnaire, VVIQ)^58^.

This Cyberball experiment followed a well-established protocol^32,37,42^. Participants were informed that they would play a virtual ball-tossing game with two other co-players who were connected to them via the internet. Participants viewed three dots displayed on the screen, each displayed for 2 s ± 12ms, to simulate the process of connecting to the Internet. The following screen, with a resolution of 1280 × 1024 pixels, displayed a line drawing of the two putative co-players and the avatar of their choice. To increase participant involvement and sense of control, participants were asked to choose their own avatar to represent themselves at the beginning of the game from six different options^42^.

The actual game screen is illustrated in Fig. 1a. At the viewing distance of 120 cm, the entire stimulus displays of the game subtended 7° × 7° degrees of visual angle. The avatar of the participant was located 5° below the central point of the computer screen, and the two avatars assigned to two co-players were each located 2.5° to the left and right of the centre, respectively. To control for potential extraneous variables that could affect the results of the study (e.g., excessive saccades or micro saccades), the eye gaze direction of the avatars was maintained consistently throughout each block. The motion of the ball’s trajectory as it was being passed from one player to another was not shown. Instead, the ball simply appeared once near the sender’s avatar and subsequently near the receiver’s avatar, which helped to reduce eye movements.

Participants were instructed to mentally visualize the game in detail and were informed that they were playing with other players, consistent with the cover story. However, they were not explicitly informed about the gaze choices of the avatars. This approach allowed participants to interpret the eye gaze direction of the avatars as indicative of the co-players’ social intentions, leveraging the human tendency to use eye gaze as a non-verbal cue in social interactions. For that purpose, a picture of a meadow or a beach was shown before each experimental block, along with an instruction to imagine the ball tossing game taking place there. During the game, ball possession was signalled by the ball appearing next to the avatar. The participant was told to pass the ball to either the left or the right co-players by pressing the corresponding keyboard button (left or right arrow key on a keyboard). After a player pressed the arrow key, the ball disappeared for 500 ms and then reappeared next to one of the co-players. To simulate the variability in the co-players’ decision making, the co-players held the ball for a randomized duration between 400 and 1400 ms before making a toss.

For eye gaze manipulation, we employed black line drawings on a white background, akin to the study of Friesen & Kingston (2003)^59^. In the first block, all depicted faces exhibited a forward-facing gaze. In the second block, the gaze direction varied, being either downward, leftward-averted, or rightward-averted. Pertaining to the ‘Gaze Direction’ factor, one group was presented with faces showing a neutral expression and a downward gaze towards the participant’s avatar (referred to as the ‘direct gaze’ group), while the other group observed faces with gaze averted towards the co-player’s avatars (termed the ‘averted gaze’ group), as illustrated in Fig. 1a. Throughout each of Block 1 and Block 2, the avatars’ gaze was kept consistent to mitigate involuntary ocular EEG artifacts from (micro)saccades, which could arise from transient gaze alterations^60,61^.

Following written instructions and a brief 10-trials training session with 33% ball reception probability, participants engaged in the primary tasks: two blocks of the Cyberball game. Each block consisted of 200 ball throws and lasted approximately 7 min, bringing the total duration of the experiment around 20 min. In the first block, the avatars displayed a neutral forward-looking gaze, and the participant received the ball in approximately one-third of all throws (fair proportion of 33%, approx. 66 times). In the second block, the avatars displayed either an averted gaze or displayed a direct gaze, see Fig. 1a. The probability of receiving the ball was reduced to 23% (approx. 46 times) in the second block. It is noteworthy that a ball reception rate of 23% was chosen to induce a moderate level of exclusion. Typically, a ball reception rate of 16% is used in a partial exclusion setup. To avoid ceiling effects potentially masking the impact of gaze cues, we chose a 23% reception rate, aligning with Hartgerink et al. (2018) and Schuck et al. (2018), to induce a moderate yet sufficient level of exclusion for exploring gaze behaviour’s nuanced effects. In both blocks, participants started with possession of the ball.

After completing the second block, participants completed three short online questionnaires: the NTQ, the negative mood scale^62^, and a ball reception estimation. The NTQ assessed changes in fundamental social needs across four dimensions: belonging, self-esteem, meaningful existence, and control. Each dimension included three items (e.g., “I felt disconnected” for belonging, “I felt non-existent” for meaningful existence). Responses were collected on a seven-point scale ranging from − 3 (“Much Stronger in Block 1”) to 3 (“Much Stronger in Block 2”), focusing on changes from inclusion to exclusion phases. For the negative mood scale, participants rated eight items from the Positive and Negative Affect Schedule (PANAS) questionnaire (e.g., “I felt angry”). Scores were calculated by averaging responses, with the final score (range: −12 to + 12) indicating the magnitude and direction of mood change, where negative scores reflected stronger negative mood during inclusion. Lastly, participants were asked to estimate separately the frequency of receiving the ball from their co-players in Block 1 and Block 2. After completing the questionnaires, participants were adequately debriefed about the real purpose of the study and provided written informed consent again.

### EEG recording and pre-processing

For electrophysiological measurements, we used the same setup as in previous studies from our lab^42^ to ensure consistency and comparability. A BrainAmps amplifier (BrainProducts, Gilching, Germany) was used to acquire the EEG signals from eight active electrodes positioned at standard 10–20 system locations (AFz, Fz, F3, F4, Cz, Pz, P7, and P8) within an elastic cap (EASYCAP, Herrsching, Germany). All channels were referenced online to the electrodes located on the left and right mastoids, with impedance levels kept below 10 kΩ. The ground electrode was placed at FCz. In addition, to control for ocular artifacts, data was recorded from four electrooculogram (EOG) electrodes placed near the canthus of the left and right eye as well as above and below the right eye. All EEG and EOG data were digitised at a rate of 500 Hz and processed filtered online with a bandpass filter between 0.1 and 100 Hz.

The raw EEG data underwent preprocessed using Brain Vision Analyser (version 2.1, Brain Products, Gilching, Germany). The data were subjected to an offline bandpass-filtered using a Butterworth filter (0.3 to 30 Hz, 24 dB/Oct)^37,42^ and segmented relative to the onset of the ball reception event of the participants (− 100 to 700 ms). Subsequently, bipolar EOG channels were calculated offline by subtracting the left canthus from the right canthus electrode (bipolar horizontal EOG, HVOG) and the subraorbital from the infraorbital EOG electrode (bipolar vertical EOG, VEOG). The ERP epoch was adjusted by subtracting average channel voltages during a 100 ms pre-stimulus baseline (from − 100 to 0 ms). Afterwards, trials with excessive ocular artifacts (V/HEOG > 50 µV) in a single segment were identified and excluded from the analysis. Epochs with amplitudes surpassing a threshold of ± 80 µV in active electrodes were flagged using a semiautomatic artifact detection. These flagged trials were then manually reviewed for slow movement-related artifact (linear drifts) and high frequent bursts. Additionally, participants with an average ERP signal based on fewer than 20 segments in each block were removed from the analysis. Owing to these stringent rejection criteria, the final average ERP in this study was based on a mean of 38.6 segments for Block 1 (with an average of 27.4 excluded trials) and 23.9 for Block 2 (with an average of 22.1 excluded trials) were. It is important to highlight that the number of remaining epochs was lower in Block 2 due to the decreased likelihood of ball reception in this social exclusion block.

### Data analysis

#### Questionnaire data

Data from the estimated ball reception, NTQ, and the negative mood were analysed for each participant (R Core Team, 2022, version 4.1.2).

##### Ball reception

Firstly, a 2 (Block: block 1 vs. block 2) × 2 (Gaze: averted vs. direct gaze) analysis of variance (ANOVA) was conducted to examine the estimated frequency of participants’ ball reception and to assess the impact of the experimental manipulation. The results of the ANOVA are reported with Greenhouse-Geisser corrected degrees of freedom and *p*-values, and Cohen’s d as a measure of effect size, and 95% confidence intervals (95% CI). In case of significant interactions of the experimental factors, the corresponding Bonferroni-Holm method was used to correct all post-hoc tests.

##### Need-threat questionnaire

To comprehensively assess the effects of eye gaze manipulation on the scores across the four NTQ scales (belonging, self-esteem, meaningful existence, and control). All items were assessed on a 7-point scale (ranging from − 3, “Much Stronger in Block 1” to 3, “Much Stronger in Block 2”) requiring a relative judgment. Participants rated the extent to which their feelings were more expressed in block 1 compared to block 2, or vice versa. This relative rating approach allows us to focus on the changes in feelings between blocks, rather than requiring independent judgments for each block. Therefore, negative values in our results indicate stronger feelings during block 1, while positive values indicate stronger feelings during block 2. The final scores maintain this − 3 to + 3 range, directly reflecting the relative strength of feelings between blocks^36,39,42^. We employed a mixed-design Multivariate Analysis of Variance (MANOVA). First, the internal consistency for the subscales of NTQ was evaluated using Cronbach’s alpha. The results indicated satisfactory reliability across all dimensions: belonging (α = 0.65), self-esteem (α = 0.67), meaningful existence (α = 0.54), and control (α = 0.74). These values confirm the scales’ adequacy for assessing the psychological impact of social exclusion, consistent with prior research^42^. This approach enabled examination of the influence of eye gaze direction on social exclusion processing, as reflected by the NTQ scales. Bonferroni post-hoc corrections were applied as necessary to control for multiple comparisons. This method was selected to account for multiple comparisons while assessing the differential impact of the experimental manipulation on each psychological construct measured by the NTQ.

##### Negative mood

For the analysis of negative mood, which was assessed as a single item, an independent t-test was conducted to compare the negative mood scores between the direct and averted gaze groups. The results are presented as t-values, *p*-values, Cohen’s d effect sizes, and 95% CI. The threshold for statistical significance was set at *p* < .05.

### EEG data

The relevant time-locking event for all ERP analyses was the player receiving the ball from one of the co-players. While we also analyzed co-player passing events (see Supplementary Materials, “Analysis of the P3 responses for co-players” section), these events did not elicit reliable P3 responses. Therefore, we focused our main analysis on ball reception events as they provided more robust markers of social exclusion processing. Event-related potentials were averaged separately for each participant and condition and analysed according to the between-subject factor “Gaze direction” (direct gaze, averted gaze) and the within factor “Block” (33%, 23%). The ERP epoch, spanning from − 100 ms pre ball reception to 700 ms post ball reception, ensured no overlap with the previous play’s ERP activity.

We initially utilized the Global Field Power (GFP) approach to gain an overview of the P3 component. GFP quantifies the standard deviation of potentials across all electrodes at a given time point, serving as a robust metric to identify periods of heightened neural activity. This approach revealed two peaks in the P3 component. We chose to focus on the second time window from 320 to 400 ms as a predetermined interval of interest. This decision was based on prior research indicating that this time window is relevant for processing social exclusion. Our analyses were aligned with our hypotheses, allowing us to directly compare our results with established findings on transitions to social exclusion. For the analysis and results pertaining to the first time window, please refer to the Supplementary Materials section - “Early time window (262–342 ms)”.

The mean P3 amplitude for both time windows at the fronto-central ROI (Fz, Cz, F3, F4) ) was analysed in the R environment for scientific computing (R Core Team, 2022, version 4.1.2. The tidyvers^63^, afex^64^ and lsmeans packages were used for data processing and statistical analyses (ANOVA and post-hoc analysis). While P3 is traditionally associated with parietal sites, our focus on fronto-central channels was motivated by previous Cyberball studies showing robust social exclusion effects in these regions^21,37^. Additional analysis including Pz with our fronto-central ROI showed similar effects, supporting the reliability of our findings (see Supplementary Materials “Electrode homogeneity & additional channel analyses” section for details). A significance level of 5% was used to determine statistical significance. All analytical scripts and data will be made available at OSF (https://osf.io/wkrac).

To answer the first hypothesis (effects of gaze and exclusion on P3 amplitude), repeated measures 2 × 2 ANOVAs were calculated for the P3 amplitudes in the fronto-central on the between-participant factors “Gaze Direction” (averted gaze, direct gaze) and the within-participant factor “Block” (33% vs. 23%). The specific electrodes were chosen as the fronto-central ROI based on previous studies that have shown their involvement in processing social exclusion in this context^37,42^. Additionally, to examine whether gaze direction influenced P3 modulation during exclusion, we computed block differences (Δ(Block 2: exclusion - Block 1: inclusion)) and compared groups using an independent-samples t-test. This approach allowed us to assess whether direct and averted gaze differentially shaped the overall P3 response to social exclusion.

To ensure the homogeneity of the electrodes, a 2 (“Gaze Direction”) × 2 (“Block”) × 4 (“Electrodes”) ANOVA was also performed (see Supplementary Materials, “Electrode homogeneity” section). In all repeated measures ANOVAs with more than one degree of freedom, ANOVA results were reported with a Greenhouse-Geisser correction of the degrees of freedom to adjust for possible violations of the sphericity assumption (in case of a significant Mauchly test, *p* < .05). A priori significance level of 5% was used for all statistical tests and partial eta squared (*η*_*p*_^2^) was reported as measures of effect size. The Bonferroni-Holm method was used to adjust for multiple comparisons only when the effect or interaction was significant (*p* < .05). This method is a step-down multiple comparison procedure that provides more stringent control of the familywise error rate compared to other correction methods. When significant effects or interactions were found, post-hoc tests were run, and the mean (*M*) and 95% confidence interval (CI) were reported to provide further information on the results.

Our second hypothesis focused on the adaptation patterns of P3 amplitude changes within the blocks of the experimental, when exclusion is experienced (“Block”). A split-analysis was used to compare the average ERP response to the first ten (first half) and the final ten (second half) trials within each experimental block. This method, though conservative, has been successfully applied in previous Cyberball studies to detect systematic amplitude fluctuations over time^21,39,42^.

The subgroup sizes for the split analysis were determined by stringent EEG data preprocessing criteria, resulting in *n* = 16 for the averted gaze group and *n* = 23 for the direct gaze group. This selection was based on the requirement of a minimum of 20 artifact-free trials per participant per block, as endorsed by prior research^21,39,42^, ensuring data quality and consistency. Our adherence to this criterion is detailed in our methodology, emphasising the criticality of high-quality data for robust ERP analysis.

Despite the unbalanced subgroup sizes, we addressed potential statistical concerns by employing analytical techniques tailored for EEG data. Specifically, our analysis considered ‘group’, ‘block’, and ‘half’ as fixed effects, while accounting for subjects nested within these factors as random effects. This approach, designed for handling within-subject variability and unbalanced group sizes, mitigates heteroscedasticity, and maintains statistical power by using Type III sum of squares and partial eta squared as an effect size measure. Notably, the findings from this subgroup analysis were consistent with the primary results derived from the entire participant sample. The mean amplitudes in the time range of the P3 components were calculated, followed by a 2 (between-subject factor: “Gaze direction”: averted-gaze, direct-gaze) × 2 (within-subject factor: “Block”; inclusion vs. exclusion) × 2 (within-subject factor " Split”: 1st vs. 2nd half) ANOVA. The factor ‘Electrode” was not included (see above). To specifically investigate the adjustment process, we focused our analysis on Block 2 and conducted a 2 (“Gaze direction”: averted-gaze, direct-gaze) × 2 (“Split”: 1st vs. 2nd half) ANOVA, examining the mean amplitudes within the late P3 time window. This analysis was designed to detect potential temporal adaptation effects related to gaze direction. The key statistical results are presented in the Results section.

## Electronic supplementary material

Below is the link to the electronic supplementary material.

Supplementary Material 1

## Data Availability

All analytical scripts and data will be made available at OSF (https://osf.io/wkrac).
