# Supplementary material for: Direct and averted gaze modulate the event-related brain responses to social exclusion signals
Source: Sci Rep. 2025 Apr 18;15:13396. doi: 10.1038/s41598-025-97840-4 (PMC12008228; doi:10.1038/s41598-025-97840-4)
Supplement: Supplementary file 1 — Supplementary Material 1 [file 41598_2025_97840_MOESM1_ESM.pdf]

Supplementary

Direct and averted gaze modulate the event-related brain responses to social exclusion signals

Yu-Fang Yang*[1], Xu Fang[1], & Michael Niedeggen[1]

1. Division of Experimental Psychology and Neuropsychology, Department of Education and Psychology, Freie Universität Berlin, Berlin, Germany

ERP results

Early time *window* (262-342 ms)

The first time window was identified based on the peak Global Field Power (GFP) and spanned from 262 to 342 ms. This P3 component was discerned at frontal-central electrode locations with a peak latency of 302 ms. Consistent with prior research (Niedeggen et al., 2017; Schuck et al., 2018), the analysis was confined to an 80 ms window centered on this estimated peak latency, offering a ± 40 ms range.

The amplitude of the early positivity was more positive during the second block (i.e., exclusion) compared to the first block (i.e., inclusion), indicating that the transition to exclusion increased the amplitude of the early positivity, $F(1,54) = 9.23$, $p < .01$, $\eta_p^2 = .146$. The amplitude in block 2 ($M = 4.78$ µV, 95% CI [4.02, 5.53]) was greater than in block 1($M = 3.74$ µV, 95% CI [2.91, 4.57]). Although the increase appeared to be more pronounced in the direct gaze group, the ANOVA did not reveal a significant main effect of the group, $F(1,54) = 0.9$, $p = .346$, $\eta_p^2 = .011$, or a significant interaction between the factors 'Group' and 'Block', $F(1, 54) = 2.36$, $p = .130$, $\eta_p^2 = .044$.

Split-half analysis: adaptation effects within blocks

The magnitude of increase in P3 amplitudes within the second block ($M = 5.11$ µV, 95% CI [4.00, 6.19]) was significantly greater than that observed within the first block ($M = 3.74$ µV, 95% CI [2.47, 5.00]), $F(1,38) = 9.38$, $p = .004$, $\eta_p^2 = .198$. This result is consistent with the previous analysis that included all subjects. No significant main effect was observed for the "Group", $F(1,38) = 0.83$, $p = .368$, $\eta_p^2 = .021$, or for the "*Split*", $F(1,38) = .21$, $p = .646$, $\eta_p^2 = .006$. Moreover, a significant interaction emerged among group, block, and split, $F(1, 38) = 8.71$, $p = .005$, $\eta_p^2 = .186$, which allowed separated analyses for the effects of 'group' and 'split-half' within the two blocks. In the first block, the P3 amplitude remained stable for both

experimental groups, as evidenced by the absence of a significant main effect for the group ($F(1,38) = .03, p = .865, \eta_p^2 < .001$), and split ($F(1,38) = .58, p = .451, \eta_p^2 = .015$). Furthermore, no significant interaction was observed between the group and split, $F(1, 38) = 1.45, p = .235, \eta_p^2 = .037$.

In the second block, the P3 amplitudes showed a gradual reduction in the direct gaze group, while increasing in the averted gaze group. The ANOVA results did not reveal significant main effects for the factor group, $F(1,38) = 2.8$, $p = .103$, $\eta_p^2 = .069$, or a main effect of the factor split, $F(1,38) = .02, p = .892, \eta_p^2 < .001$. However, a significant interaction between these factors was observed, $F(1,38) = 8.24, p = .007, \eta_p^2 = .178$. The subsequent post-hoc comparison demonstrated that the amplitude of P3 was marginally significantly higher in the direct gaze group ($M = 6.52$ µV, 95% CI [4.95, 8.07]) compared to the averted gaze group ($M = 3.36$ µV, 95% CI [1.66, 5.06]) in the first split of the block ($p = .05$).

Electrode homogeneity & additional channel analyses

To ensure the homogeneity of the electrodes, a 2 ("Group") x 2 ("Block") x 4 ("Electrodes") ANOVA was performed. Here are the results for both early and late time windows.

Early time window

In the 262 ms – 342 ms time window, the main effect of Group was not significant, $F(1, 54) = 0.90$, $p = .346$, $\eta_p^2 = .016$. The main effect of Block was significant, $F(1, 54) = 9.23$, $p = .004$, $\eta_p^2 = .146$. Furthermore, the main effect of Electrodes was significant, $F(1.75, 94.55) = 47.64$, $p < .001$, $\eta_p^2 = .042$. The interaction effect of Group × Block was not significant, $F(1, 54) = 2.36$, $p = .130$, $\eta_p^2 = .042$. The interaction effect of Group × Electrodes was also not significant, $F(1.75, 94.55) = 0.20$, $p = .793$, $\eta_p^2 = .004$. The interaction effect of Block × Electrodes was significant, $F(2.39, 128.81) = 4.30$, $p = .011$, $\eta_p^2 = .074$. Lastly, the interaction effect of Group × Block × Electrodes was not significant, $F(2.39, 128.81) = 1.63$, $p = .194$, $\eta_p^2 = .029$.These results indicate that the chosen fronto-central electrodes are homogeneous across the different conditions.

Late time window

In the 320ms - 400 ms time window, we found no significant main effect of Group, $F(1, 54) = 2.00$, $p = .163$, $\eta_p^2 = .036$, suggesting that the group differences did not significantly

impact P3 amplitudes. The main effect of Block was significant, $F(1, 54) = 20.61, p < .001, \eta_p^2 = .276$, indicating a clear distinction in P3 amplitudes between the two blocks. Furthermore, the main effect of Electrodes was also significant, $F(1.74, 94.17) = 42.77, p < .001, \eta_p^2 = .442$, validating the involvement of the selected fronto-central electrodes (F3, F4, Cz, Pz) in processing social exclusion within this timeframe.

Significant interactions were observed: the interaction of Group × Block, $F(1, 54) = 5.23$, $p = .026$, $\eta_p^2 = .088$, underscores the differential effects of social exclusion across groups. However, the Group × Electrodes interaction did not reach significance, $F(1.74, 94.17) = 0.20$, $p = .786$, $\eta_p^2 = .004$, suggesting uniform electrode responsiveness across groups. The Block × Electrodes interaction was significant, $F(2.49, 134.44) = 5.05$, $p < .01$, $\eta_p^2 = .086$, highlighting the variable impact of exclusion across electrode sites. Critically, the absence of a significant three-way interaction between Block, Electrodes, and Group, $F(2.49, 134.44) = .55$, $p = .616$, $\eta_p^2 = .01$, indicates that the specific impact of social exclusion, as modulated by gaze direction, did not differentially manifest across the chosen electrode sites. This finding suggests a generalizable pattern of neural responsiveness to social exclusion cues across the fronto-central region, irrespective of gaze direction. These results support the electrode selection's appropriateness for capturing relevant neural signatures of social exclusion across conditions.

Electrode placement checks

Fronto-central site. To address potential concerns regarding electrode placement and ensure the integrity of our findings, we further conducted a focused examination of the electrode responses, clustering them into fronto-central (1st cluster: F3, F4) and central (2nd cluster: Cz, Pz) groups. This analysis utilised a 2 (Cluster) x 2 (Block) x 2 (Group) ANOVA framework. The results, as depicted in the attached figure, indicated a significant main effect of Block, $F(1, 54) = 13.95$, $p < .001$, $\eta p2 = .205$, demonstrating a noticeable difference in P3 amplitudes between the two blocks across clusters. The interaction effects involving the clusters were not significant: Group × Cluster, $F(1, 54) = 0.21$, $p = .648$, $\eta p2 = .004$; Block × Cluster, $F(1, 54) = 0.33$, $p = .600$, $\eta p2 = .005$; and the three-way interaction Group × Block × Cluster, $F(1, 54) = 0.28$, $p = .600$, $\eta p2 = .005$. This lack of significant interaction underscores the homogeneity of the electrode response within these clustered regions, lending further support to the consistency and reliability of our electrode placement strategy in detecting neural markers of social exclusion. These findings bolster our confidence in the methodological

design of our study and suggest that the fronto-central and central clusters are comparably responsive in the context of social exclusion processing within our experimental setup.

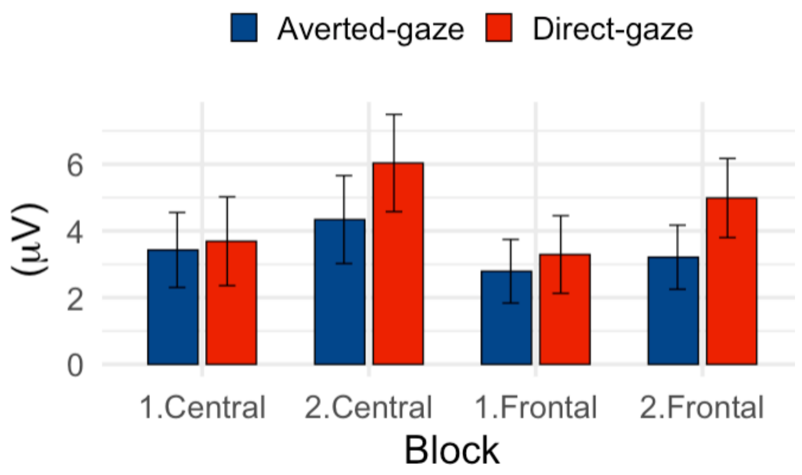

FigureS1: Mean P3 amplitude responses at fronto-central (F3, F4) and central (Cz, Pz) electrode clusters. Error bars represent 95% CI.

Parietal site. To validate our channel selection, we conducted additional analyses examining the contribution of parietal sites to the P3 component during the same time window (320-400 ms). The analysis incorporating Pz into our fronto-central ROI (F3, F4, Cz, Fz, Pz) revealed consistent patterns with our original findings. A 2 (Group) × 2 (Block) repeated-measures ANOVA showed a significant main effect of Block ($F(1, 54) = 19.94$, $p < .001$, $\eta_p^2 = 0.27$), with higher amplitudes in Block 2 (M = 5.17 µV, 95% CI [4.32, 6.02]) compared to Block 1 (M = 3.89 µV, 95% CI [3.10, 4.68]). A significant Group × Block interaction emerged ($F(1, 54) = 4.48$, $p = .039$, $\eta_p^2 = 0.077$), with post-hoc tests revealing a significant increase in P3 amplitude from Block 1 to Block 2 specifically in the direct gaze group ($t(54) = 4.655$, $p = .0001$). No significant main effect of Group was observed ($F(1, 54) = 1.98$, $p = .165$).

Analysis of the P3 responses for co-players

To further elucidate the potential influence of gaze cue informativeness on the observed P3 effects, we conducted an additional analysis on the P3 responses of co-players across two distinct time windows: 264-342ms and 320-400ms.

Early time window

264-342ms*:* The amplitude of the early positivity did not differ significantly between the two gaze groups, $F(1, 54) = 0.71$, $p = .404$, $\eta_p^2 = .013$ (See Figure S2). The transition

between blocks did not result in a significant change in the amplitude, $F(1, 54) = 2.53$, $p = .118$, $\eta_p^2 = .045$. Furthermore, the interaction between gaze group and block was not significant, $F(1, 54) = 0.05$, $p = .826$, $\eta_p^2 = .001$. This suggests that the early positivity amplitude remained consistent across gaze groups and blocks.

Late time window

320-400ms*:* The amplitude of the late positivity did not show a significant difference between the two gaze groups, $F(1, 54) = 0.03$, $p = .860$, $\eta_p^2 = .001$(See Figure S2). However, there was a significant main effect of block, $F(1, 54) = 5.43$, $p = .024$, $\eta_p^2 = .091$. This indicates a statistically significant difference in the amplitude of the late positivity between the two blocks. The interaction between gaze group and block was not significant, $F(1, 54) = 1.41$, $p = .241$, $\eta_p^2 = .025$.

Electrode homogeneity

To further validate our stance, we conducted an ANOVA to ensure electrode homogeneity. Specifically, a 2 (”Groups”) x 2 (”Block”) x 4 (“Electrodes”) ANOVA was executed. For the time window of 264-342ms, the analysis did not manifest any significant Group or Block differences, nor any Group and Block interactions. Similarly, for the time window of 320-400ms, the results were consistent with no significant Group or Block differences or interactions. This further corroborates our stance that the P300 differences are predominantly influenced by the embodied effect of gaze direction rather than cue informativeness.

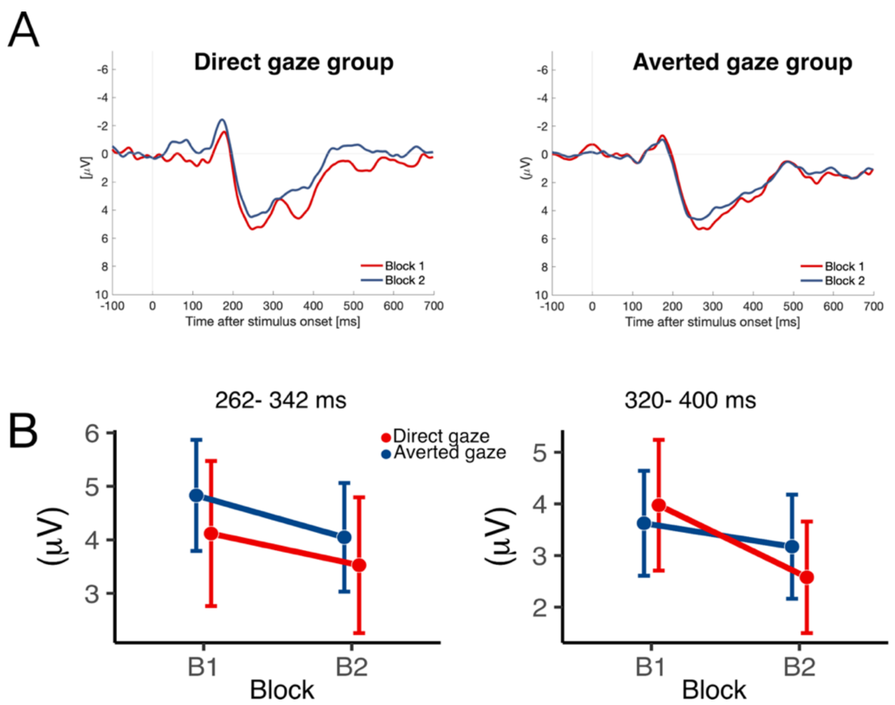

Figure S2: Event-Related Potential (ERP) Analysis of Coplayer Receiving Ball Event. **A)** Grand-average ERP waveforms from a central region of interest (comprising Fz, Cz, F3, F4 electrodes) synchronized to the onset of the coplayer receiving the ball. The waveforms are differentiated by block (Block 1 depicted in red; Block 2 in blue) and by gaze group (direct gaze and averted gaze). **B)** Line plots showcasing P3 amplitude variations across Fz, Cz, F3, and F4 channels for both Block 1 and Block 2. Comparisons are made between the direct gaze group (illustrated in red) and the averted gaze group (shown in blue) within two distinct time windows: 262-342 ms and 320-400 ms. Error bars represent the 95% CI.

The results from this supplementary analysis suggest that the observed P300 disparities in our primary study are not driven by cue informativeness. This aligns with our primary findings and further emphasizes the psychological implications of direct versus averted gaze in the context of social exclusion.
